# Comparison of full-length versus non-full-length vaginal tightening for moderate-to-severe vaginal laxity syndrome: A retrospective cohort study

**DOI:** 10.1097/MD.0000000000050051

**Published:** 2026-08-14

**Authors:** Fengmei Li, Chunyan Song

**Affiliations:** a Department of Obstetrics and Gynecology, Zhengzhou Central Hospital Affiliated to Zhengzhou University, Zhengzhou, Henan, China.

**Keywords:** full-length reconstruction, pelvic floor, sexual function, vaginal laxity syndrome, vaginal tightening

## Abstract

Vaginal laxity syndrome (VLS) is a common pelvic floor disorder that compromises sexual function and quality of life. Although full-length vaginal tightening has been proposed as a more comprehensive approach than conventional non-full-length techniques, direct comparative evidence using multidimensional outcome measures remains scarce. This study aimed to compare the efficacy and safety of full-length versus non-full-length vaginal tightening procedures in patients with moderate-to-severe VLS. This retrospective cohort study included 100 patients with moderate-to-severe VLS who underwent surgery between January 2024 and May 2025 at Zhengzhou Central Hospital. Patients were divided into a control group (non-full-length vaginal tightening, n = 50) and an observation group (full-length vaginal tightening, n = 50). The primary outcomes included subjective sexual function assessments (vaginal laxity questionnaire [VLQ], sexual satisfaction questionnaire, female sexual function index, and female sexual distress scale-revised) and objective anatomical and physiological measures (vaginal capacity, levator hiatus area, genital hiatus, perineal body, and pelvic floor electromyography). Secondary outcomes included operative time, blood loss, hospital stay, and complications. Assessments were conducted preoperatively and at 3, 6, and 12 months postoperatively. The full-length group demonstrated significantly greater improvements in VLQ, sexual satisfaction questionnaire, female sexual function index total score, and female sexual distress scale-revised scores compared with the non-full-length group (all *P* for interaction <.05). Although both groups achieved a postoperative vaginal capacity of 2 fingerbreadths with no significant between-group difference (*P* = .595), the full-length group exhibited significantly higher VLQ scores (*P* = .008). The full-length group also showed significantly greater reductions in levator hiatus area and genital hiatus, and greater improvement in fast-twitch muscle contraction amplitude (all *P* for interaction <.05). No significant between-group difference was observed in perineal body improvement (*P* = .120). Operative time and blood loss were slightly higher in the full-length group (98.33 vs 92.73 minutes; 27.10 vs 23.88 mL, both *P* < .05), but these differences did not translate into increased complication rates or prolonged hospital stay. For patients with moderate-to-severe VLS, full-length vaginal tightening achieves more comprehensive anatomical reconstruction and functional restoration of pelvic floor support structures, significantly improves sexual function and quality of life, and does not incur additional surgical risks. Full-length vaginal tightening can be considered a preferred surgical option for moderate-to-severe VLS.

## 1. Introduction

Vaginal laxity syndrome (VLS) is a common pelvic floor disorder resulting from structural damage to the pelvic floor sustained during pregnancy and childbirth. It is characterized predominantly by an increase in vaginal caliber, which compromises contractile strength and sensitivity; in severe cases, this can be further complicated by stress urinary incontinence, pelvic organ prolapse, and sexual dysfunction.^[1,2]^

With the growing emphasis on quality of life and sexual well-being, function-oriented vaginal tightening procedures have evolved considerably.^[3]^According to DeLancey 3-level theory of vaginal support, VLS involves compromised structural support across all 3 levels (levels I–III).^[4]^For moderate-to-severe VLS, although a variety of surgical techniques have been introduced, the central focus of most remains on tightening the lower 3rd of the vagina, aiming to narrow the vaginal introitus and reinforce the levator ani muscle and perineal body.^[5]^

Currently, however, there is little consensus regarding the choice of procedure, the extent of surgical repair, and the criteria for outcome evaluation.^[6]^ Evidence indicates that such non-full-length techniques, despite their widespread use, are associated with insufficient improvement in the sensation of tightness in the middle and upper vagina and with long-term dyspareunia in a subset of patients,^[7,8]^ underscoring the limitations of repairing level III alone. Extending the repair to the full length of the vagina would theoretically allow more comprehensive functional reconstruction of the vaginal axis and the pelvic floor support apparatus.^[9]^ The concept of full-length vaginal tightening has been proposed, and preliminary investigations have demonstrated its clinical potential.^[10,11]^ Nevertheless, the vast majority of existing evidence derives from outcome descriptions of single techniques, and there is a paucity of high-level evidence directly comparing full-length and conventional non-full-length approaches using multidimensional outcome measures. To address this gap, this retrospective cohort study was designed to systematically compare full-length versus conventional non-full-length vaginal tightening procedures with respect to subjective sexual function, objective pelvic floor anatomical and functional recovery, and surgical safety, thereby providing evidence to guide the choice of surgical extent in clinical practice.

## 2. Methods

### 2.1. Patients

This retrospective study reviewed the medical records of patients with VLS who underwent surgery at the Zhengzhou Central Hospital between January 2024 and May 2025. The inclusion criteria were: diagnosis of moderate or severe VLS based on the Guidelines for Plastic Surgery Treatment of Vaginal Laxity^[1]^; presence of surgical indications for vaginal tightening; provision of written informed consent before surgery; normal mental state and cognitive function; and complete clinical data. The exclusion criteria were: concurrent acute urogenital infections or pelvic malignancy; coagulation disorders or other hematologic or immunologic diseases; prior pelvic floor surgery or pelvic radiotherapy; pelvic organ prolapse of pelvic organ prolapse quantification (POP-Q) stage II or above; severe sexual dysfunction unrelated to VLS; and cognitive impairment or consciousness disturbance. The study protocol was approved by the hospital ethics committee (approval number: ZXYY2025167), and written informed consent was obtained from all participants. Based on the extent of surgery, 100 patients were divided into a control group (non-full-length vaginal tightening, n = 50) and an observation group (full-length vaginal tightening, n = 50). All procedures were performed by a fixed, dedicated surgical team to ensure procedural consistency.

### 2.2. Surgical intervention

#### 2.2.1. Control group

In the control group (non-full-length tightening), surgical manipulation was confined to the lower 3rd of the vagina. After marking the 3 and 9 o’clock positions of the hymenal remnants, the rectovaginal space was dissected to the marked level, exposing the rectal fascia and levator ani. The rectal fascia was closed with interrupted 5-0 Vicryl sutures, and 3 interrupted 3-0 Vicryl sutures were placed in the levator ani to reinforce the pelvic floor. The posterior vaginal mucosa was then anchored to the levator surface and continuously sutured to the inner hymenal ring.

#### 2.2.2. Observation group

In the observation group (full-length tightening), the dissection was extended cranially to the level of the external cervical os, enabling a full-length repair. Starting at the cervix, U-shaped 2-0 Vicryl sutures were placed along the rectal fascia to depress the anterior rectal wall; the levator ani was sutured in the same fashion. The vaginal mucosa was plicated and fixed to the pelvic floor structures with 3-0 Vicryl. To achieve progressive narrowing from the apex to the introitus, bilateral levator and mucosal sutures were placed continuously down to the hymenal ring, where the mucosal flap was folded and secured.

All procedures were performed 3 to 10 days after menstruation under general anesthesia, with patients in the lithotomy position. The core technique in both groups was vaginal tightening with preservation of the vaginal mucosa and concomitant perineal body reconstruction. The perineal body reconstruction step was identical: a diamond-shaped perineal incision was made to excise redundant skin and soft tissue, after which the levator ani and superficial transverse perineal muscles were approximated to elevate the perineal body. A digital rectal examination was routinely performed to exclude injury, and the introitus was calibrated to 2-finger patency before vaginal packing with iodophor gauze. Postoperative care, including sitz baths, topical estrogen, and activity restrictions, was standardized for both groups.

### 2.3. Outcome measures

#### 2.3.1. Baseline characteristics

Demographic and clinical data included age, body mass index, menopausal status, parity, delivery mode, vaginal laxity severity, and history of perineal laceration.

#### 2.3.2. Primary outcomes

The primary efficacy endpoint was the improvement in vaginal laxity and sexual function at 3 and 6 months postoperatively, assessed through subjective and objective measures.

Subjective outcomes were assessed using a series of validated, disease‑specific and generic questionnaires that evaluated vaginal laxity, sexual function, and sexual‑related distress. Specifically, vaginal laxity was assessed using the vaginal laxity questionnaire (VLQ), a 7‑item single‑domain instrument that rates perceived vaginal looseness on a 7‑point scale ranging from 1 (very loose) to 7 (very tight), with higher scores indicating greater perceived tightness. Sexual satisfaction was measured with the sexual satisfaction questionnaire (SSQ), a 6‑point scale ranging from 1 (none) to 6 (excellent), where higher scores reflect greater satisfaction with sexual life. Overall sexual function was evaluated using the female sexual function index (FSFI), a 19‑item multidimensional instrument that assesses 6 domains: desire, arousal, lubrication, orgasm, satisfaction, and pain. The total FSFI score ranges from 2 to 36, with higher scores indicating better sexual function; a cutoff of ≤23.45 was used to define sexual dysfunction in this study. Sexual‑related distress was measured using the female sexual distress scale‑revised (FSDS‑R), a 13‑item scale that evaluates personal distress related to sexual function, with total scores ranging from 0 to 52; a score of ≥11 was considered indicative of clinically significant sexual distress. All questionnaires were self‑administered by patients in a private setting at each follow‑up visit, and the results were collected and reviewed by trained research assistants who were blinded to group allocation.

Objective outcomes comprised both anatomical and physiological assessments. Vaginal laxity grade was determined by a digital examination performed by a single senior physician who was blinded to group allocation. Vaginal capacity was evaluated as the maximum number of fingerbreadths accommodated and was classified as normal (≤2 fingerbreadths), mild (2–3 fingerbreadths), moderate (3–4 fingerbreadths), or severe (>4 fingerbreadths). Pelvic floor muscle function was assessed using surface electromyography (sEMG) according to the Glazer protocol, employing the PHENIX USB4 system (Electronic Concept Lignon Innovation). The protocol measured electromyographic amplitude (in microvolts, μV) during the resting phase, fast-twitch maximal voluntary contraction (MVC), tonic contraction, and endurance contraction phases. Levator hiatus area (LHA) was measured by 3D/4D transperineal ultrasound (Voluson E8, GE Healthcare) during the maximal Valsalva maneuver, with the area calculated in the axial plane at the level of minimal hiatal dimensions. POP-Q parameters, including genital hiatus (GH) and perineal body (PB), were measured in centimeters at maximum Valsalva using a graduated vaginal speculum. All ultrasound and POP-Q measurements were performed by the same 2 trained sonographers and gynecologists, who were blinded to the patients’ group assignments, to ensure consistency and minimize measurement bias.

#### 2.3.3. Secondary outcomes

Secondary outcomes included intraoperative parameters, postoperative recovery indicators, and procedure-related complications. Intraoperative parameters comprised operative time (measured in minutes from skin incision to completion of vaginal packing) and intraoperative blood loss (estimated by the surgical team based on suction volume and gauze weighing). Postoperative recovery was assessed by length of hospital stay (calculated from the day of surgery to the day of discharge) and postoperative pain intensity evaluated using the visual analogue scale, a 10‑point scale ranging from 0 (no pain) to 10 (worst imaginable pain), recorded at 24 hours postoperatively. Procedure‑related complications were systematically documented throughout the follow‑up period, including urinary retention (defined as the need for catheterization or inability to void spontaneously within 6 hours after catheter removal), wound hematoma, surgical site infection, delayed wound healing, rectovaginal fistula, and dyspareunia (defined as persistent or recurrent pain during or after sexual intercourse that caused distress). All adverse events were graded according to the Clavien–Dindo classification, and their management and resolution were recorded. The timing of resumption of sexual activity was also documented at each follow‑up visit, and patients were specifically queried about the occurrence and severity of dyspareunia through standardized interviews. All outcomes were assessed at baseline and at 3- and 6-month follow-up via outpatient visits, telephone, WeChat, and electronic questionnaires.

To minimize measurement bias, objective assessments, including vaginal capacity evaluation, pelvic floor ultrasound (LHA, GH, and PB), and electromyography, were performed and interpreted by senior physicians who were blinded to group allocation. All subjective questionnaires were self-administered by patients in a private setting to avoid interviewer bias.

### 2.4. Statistical analysis

In this study, the sample size was calculated mainly based on the scores of the VLQ. Referring to the results of previous studies, it was estimated that the inter-group difference in the postoperative VLQ scores between the full-segment group and the non-full-segment group was 1.0 point. Assuming a 2-sided α = 0.05 and a test power (1 − β) = 0.80, the PASS 15.0 software (NCSS, LLC) was used for calculation, and it was found that at least 36 cases were required in each group. Considering a potential data fluctuation of about 20%, it was finally decided to include 50 cases in each group.

Owing to the retrospective design and prespecified exclusion of patients with incomplete records, all enrolled patients completed the 3-, 6-, and 12-month follow-up assessments. The follow-up rate was 100%, with no missing data or loss to follow-up; thus, imputation or adjustment for missing data was not required. No sensitivity analyses were performed.

Statistical analyses were performed using SPSS 26.0 (IBM Corp.). The normality of continuous variables was assessed with the Shapiro–Wilk test. Normally distributed data are presented as mean ± standard deviation; between-group comparisons were conducted using the independent-samples *t* test, and within-group pre and posttreatment comparisons were conducted using the paired *t* test. Non-normally distributed data are expressed as median (range) and compared between groups with the Mann–Whitney *U* test, with within-group changes analyzed using the Wilcoxon signed-rank test. Categorical variables are summarized as frequencies (percentages) (n [%]) and compared with the chi-square test or Fisher exact test, as appropriate.

For repeated-measures data obtained during follow-up, normally distributed variables were analyzed by repeated-measures analysis of variance, with Greenhouse–Geisser correction applied when the sphericity assumption was violated (Mauchly test *P* < .05). Non-normally distributed repeated measures were analyzed using generalized estimating equations to evaluate the main effects of group, time, and their interaction. A 2-sided *P* < .05 was considered statistically significant.

Age, body mass index, menopausal status, and mode of delivery were prespecified as potential confounders. However, given that no significant between-group differences were observed for any baseline characteristic (Table 1), indicating satisfactory comparability, we did not perform multivariate adjustment and used univariate between-group comparisons to evaluate surgical effects.

**Table 1:** Baseline demographic and clinical characteristics of the study population.

Variable	Non-full-length group (n = 50)	Full-length group (n = 50)	*t*/χ^2^	*P* value
Age (yr)	41.2 ± 5.6	42.0 ± 6.1	0.683	.496
Body mass index (kg/m^2^)	23.5 ± 2.8	23.8 ± 3.0	0.517	.606
Menopausal status, n (%)			0.049	.825
Premenopausal	35 (70.0)	36 (72.0)		
Postmenopausal	15 (30.0)	14 (28.0)		
Parity	2 (1–3)	2 (1–3)	0.326	.744
Mode of delivery, n (%)			0.212	.899
Vaginal delivery	30 (60.0)	32 (64.0)		
Cesarean section	10 (20.0)	9 (18.0)		
Both	10 (20.0)	9 (18.0)		
Degree of vaginal laxity, n (%)			0.247	.884
Mild (3 fingerbreadths)	10 (20.0)	12 (24.0)		
Moderate (4 fingerbreadths)	30 (60.0)	28 (56.0)		
Severe (>4 fingerbreadths)	10 (20.0)	10 (20.0)		
History of perineal laceration, n (%)	22 (44.0)	20 (40.0)	0.164	.685

Non-full-length group = non-full-length vaginoplasty, Full-length group = full-length vaginoplasty. Vaginal laxity was graded by digital examination: normal, ≤2 fingerbreadths; mild, 3 fingerbreadths; moderate, 4 fingerbreadths; severe, >4 fingerbreadths (i.e., 5 or more). No patient with normal caliber (≤2 fingerbreadths) was enrolled at baseline.

## 3. Results

### 3.1. Participants

**Figure 1.**
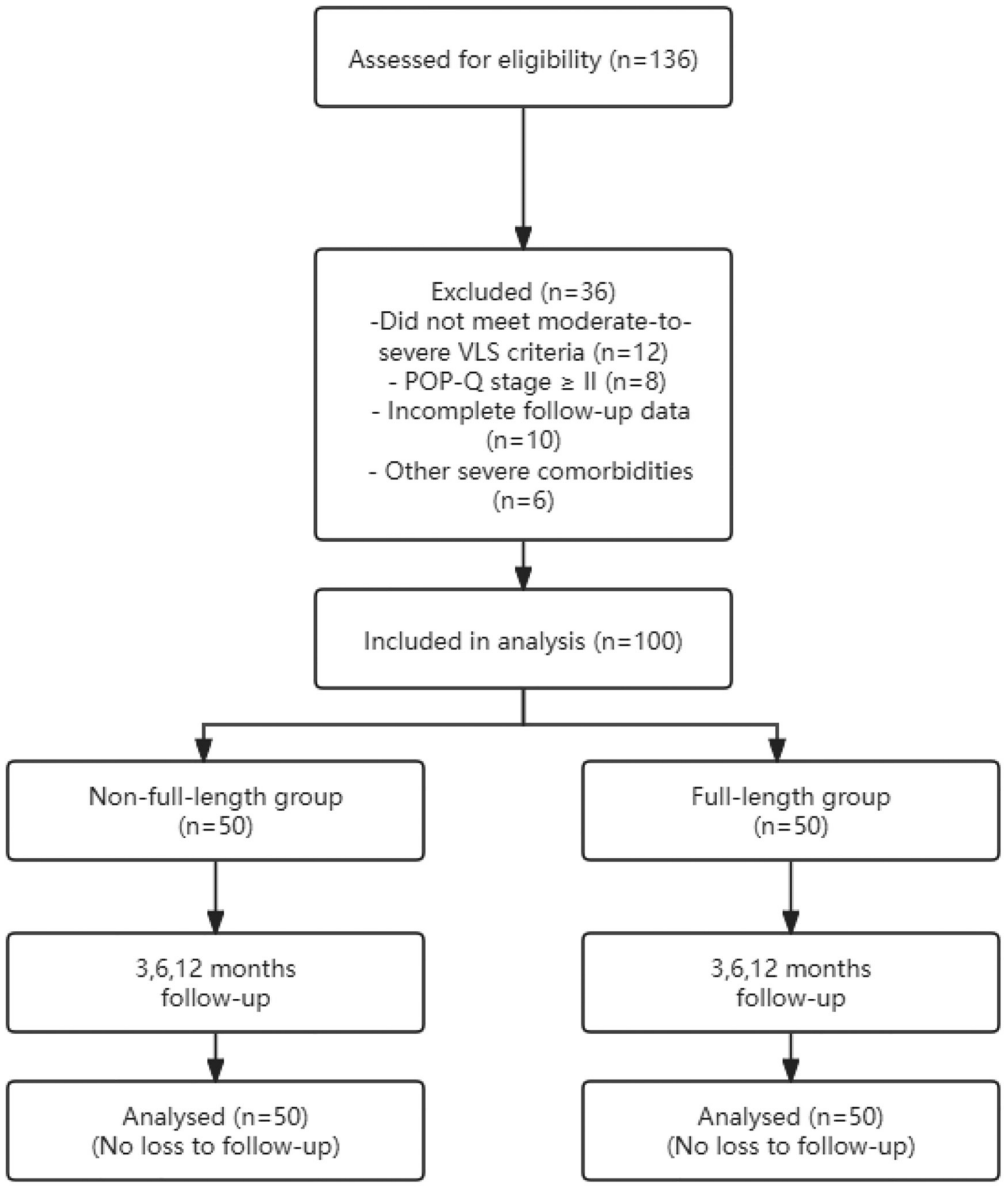
Flowchart of patient screening, enrollment, and follow-up. POP-Q = pelvic organ prolapse quantification, VLS = vaginal laxity syndrome.

A total of 136 patients were initially assessed for eligibility. After excluding 36 patients who did not meet the inclusion criteria, 100 patients were enrolled and divided into the non-full-length group (n = 50) and the full-length group (n = 50). All 100 patients completed the 3-, 6-, and 12-month follow-up assessments with no loss to follow-up (Fig. 1).

### 3.2. Baseline characteristics

A total of 100 patients were included in the analysis. Baseline demographic and clinical characteristics of the 2 groups are summarized in Table 1. No significant differences were observed between the 2 groups in terms of age, body mass index, menopausal status, parity, mode of delivery, degree of vaginal laxity, or history of perineal laceration (all *P* > .05), indicating that the 2 groups were well balanced at baseline.

### 3.3. Baseline efficacy-related parameters

Table 2 summarizes the baseline subjective questionnaire scores, objective anatomical and physiological parameters, and POP-Q measurements. No significant differences were observed between the 2 groups in any of the preoperative efficacy-related parameters, including VLQ, SSQ, FSFI total score, FSDS-R score, vaginal capacity, pelvic floor electromyography (resting amplitude, fast-twitch MVC, and slow-twitch endurance), LHA, GH, or PB (all *P* > .05), confirming the comparability of the 2 groups regarding baseline pelvic floor function and anatomy (Table 2).

**Table 2:** Comparison of baseline efficacy-related parameters between the 2 groups (mean ± SD).

Parameter	Non-full-length group (n = 50)	Full-length group (n = 50)	*t*/*Z*	*P* value
Subjective questionnaire scores				
VLQ score (1–7)	2.4 ± 0.8	2.5 ± 0.7	0.665	.507
SSQ score (1–6)	2.2 ± 0.9	2.3 ± 0.8	0.587	.558
FSFI total score (2–36)	19.0 (15.5–22.0)	19.0 (16.0–22.5)	−0.212	.832
FSDS-R score (0–52)	18.0 (14.0–22.0)	17.0 (13.0–21.5)	−0.847	.397
Objective physiological and anatomical measures				
Vaginal capacity (fingerbreadths)	4.0 (3.0–5.0)	4.0 (3.0–5.0)	−0.114	.909
Pelvic floor electromyography (μV)				
Resting amplitude	3.2 (2.6–4.0)	3.4 (2.8–4.2)	−0.576	.565
Fast-twitch MVC	27.5 ± 6.2	28.1 ± 5.9	0.496	.621
Slow-twitch endurance	19.5 (16.0–23.0)	20.0 (17.0–23.5)	−0.413	.680
Levator hiatus area on valsalva (cm^2^)	22.8 ± 3.5	22.4 ± 3.8	0.547	.585
POP-Q measurements (cm)				
Genital hiatus (GH)	4.5 ± 0.7	4.4 ± 0.8	0.665	.507
Perineal body (PB)	2.7 ± 0.6	2.8 ± 0.5	0.905	.368

Vaginal capacity was evaluated by digital examination as the maximum number of fingerbreadths accommodated.

FSDS-R = female sexual distress scale-revised, FSFI = female sexual function index, MVC = maximal voluntary contraction, POP-Q = pelvic organ prolapse quantification, SD = standard deviation, SSQ = sexual satisfaction questionnaire, VLQ = vaginal laxity questionnaire.

### 3.4. Perioperative outcomes

Perioperative indicators and postoperative complications are summarized in Table 3. The full-length group had a slightly longer operative time (98.33 ± 13.07 vs 92.73 ± 9.99 minutes, *P* = .034) and greater intraoperative blood loss (27.10 ± 7.04 vs 23.88 ± 6.76 mL, *P* = .040) compared with the non-full-length group. However, no significant differences were observed between the 2 groups in length of hospital stay or postoperative visual analogue scale pain scores (both *P* > .05). The total complication rate was 4.0% (2/50) in the non-full-length group and 6.0% (3/50) in the full-length group, with no statistically significant differences in any individual complication, including urinary retention and dyspareunia (all *P* > .05). No cases of wound hematoma, infection, delayed healing, rectovaginal fistula, or other adverse events occurred in either group.

**Table 3:** Comparison of perioperative indicators and complications between the 2 groups.

Parameter	Non-full-length group (n = 50)	Full-length group (n = 50)	*t*/*Z*	*P* value	MD/RD (95% CI)
Operative time (min)	92.73 ± 9.99	98.33 ± 13.07	−2.153	.034	5.60 (1.04–10.16)
Intraoperative blood loss (mL)	23.88 ± 6.76	27.10 ± 7.04	−2.090	.040	3.22 (0.52–5.92)
Length of hospital stay (d)	2.00 (2.00–3.00)	2.00 (2.00–2.75)	−0.316	.752	
Postoperative VAS pain score	4.00 (3.00–4.00)	4.00 (3.00–4.00)	−1.087	.277	
Complications					
Urinary retention	2 (4.0%)	2 (4.0%)	–	1.000#	
Dyspareunia	0 (0.0%)	1 (2.0%)	–	1.000#	
Wound hematoma	0 (0.0%)	0 (0.0%)	–	–	
Infection	0 (0.0%)	0 (0.0%)	–	–	
Delayed healing	0 (0.0%)	0 (0.0%)	–	–	
Rectovaginal fistula	0 (0.0%)	0 (0.0%)	–	–	
Others	0 (0.0%)	0 (0.0%)	–	–	
Total complication rate	2 (4.0%)	3 (6.0%)			

Normally distributed variables (operative time, intraoperative blood loss) are presented as mean ± SD; between-group comparisons were performed using the independent-samples *t* test. Non-normally distributed variables (length of hospital stay, VAS pain score) are presented as median (range); between-group comparisons were performed using the Mann–Whitney *U* test (*Z* statistic).Categorical variables are presented as number (%); between-group comparisons for complications were performed using Fisher exact test (#). No cases of wound hematoma, infection, delayed healing, or rectovaginal fistula occurred in either group.

CI = confidence interval, MD = mean difference, RD = risk difference, VAS = visual analogue scale.

### 3.5. Subjective outcomes

Subjective outcome measures, including VLQ, SSQ, FSFI total score, and FSDS-R score, are presented in Table 4. For all 4 subjective measures, repeated-measures analyses revealed significant group-by-time interaction effects (VLQ: *P* = .008; SSQ: *P* = .012; FSFI: *P* = .015; and FSDS-R: *P* = .009), indicating that the improvement trajectories over the 12-month follow-up period differed significantly between the 2 groups, with the full-length group demonstrating a more favorable pattern of recovery.

**Table 4:** Comparison of subjective questionnaire scores between the 2 groups before and after surgery.

Indicator	Group	Preoperative	3 mo postop	6 mo postop	12 mo postop	Time effect *P*	Group effect *P*	Interaction *P*
VLQ	Full-length	2.5 ± 0.7	5.1 ± 1.0*^,^†	5.4 ± 0.9*^,^†	5.3 ± 1.0*^,^†	<.001	.001	.008
	Non-full-length	2.4 ± 0.8	4.5 ± 0.9*	4.6 ± 0.8*	4.5 ± 0.9*			
SSQ	Full-length	2.3 ± 0.8	4.2 ± 1.1*^,^†	4.4 ± 1.0*^,^†	4.3 ± 1.1*^,^†	<.001	.005	.012
	Non-full-length	2.2 ± 0.9	3.5 ± 1.0*	3.6 ± 0.9*	3.5 ± 1.0*			
FSFI total	Full-length	19.0 (16.0–22.5)	28.5 (23.0–34.0)*^,^†	30.0 (25.0–35.0)*^,^†	29.0 (24.0–34.5)*^,^†	<.001*	0.002*	0.015*
	Non-full-length	19.0 (15.5–22.0)	25.0 (21.0–29.0)*	25.5 (22.0–30.0)*	25.0 (21.0–29.0)*			
FSDS-R	Full-length	17.0 (13.0–21.5)	7 (2–15)*^,^†	6 (0–12)*^,^†	7 (1–14)*^,^†	<.001*	.001*	.009*
	Non-full-length	18.0 (14.0–22.0)	10 (5–18)*	9 (4–16)*	10 (5–18)*			

VLQ and SSQ were normally distributed and are presented as mean ± SD; within-group comparisons used paired t-tests, and repeated measures were analyzed using RM-ANOVA. FSFI and FSDS-R were non-normally distributed and are presented as median (range); within-group comparisons used the Wilcoxon signed-rank test, and repeated measures were analyzed using GEE.

FSDS-R = female sexual distress scale-revised, FSFI = female sexual function index, SSQ = sexual satisfaction questionnaire, VLQ = vaginal laxity questionnaire.

* :*P* < .05 versus preoperative within the same group.

† *P* < .05 versus full-length group at the same time point.

* *P* values for time, group, and interaction effects for non-normal indicators are based on GEE analysis.

Within-group comparisons showed that both groups achieved significant improvements in all subjective measures at 3, 6, and 12 months postoperatively compared with the preoperative baseline (all *P* < .001). Between-group comparisons further demonstrated that the full-length group had significantly higher VLQ and SSQ scores than the non-full-length group at each postoperative time point (all *P* < .01). At 6 months postoperatively, the mean difference (MD) in VLQ score between the 2 groups was 0.8 (95% confidence interval [CI]: 0.46–1.14), and the MD in SSQ score was 0.8 (95% CI: 0.42–1.18), both favoring the full-length group. At 12 months, the corresponding MDs were 0.8 (95% CI: 0.40–1.20) for VLQ and 0.8 (95% CI: 0.36–1.24) for SSQ, both maintaining statistical significance (*P* < .01). Similarly, the full-length group exhibited a significantly greater increase in FSFI total score and a greater reduction in FSDS-R score compared with the non-full-length group (*P* for group effect = .002 and .001, respectively). At 6 months postoperatively, the median difference (Hodges–Lehmann estimate) in FSFI total score was 4.5 (95% CI: 2.0–7.0), and the median difference in FSDS-R score was −3.0 (95% CI: −6.0 to −1.0), both favoring the full-length group. At 12 months, the corresponding median differences were 4.0 (95% CI: 1.5–6.5) for FSFI and −3.0 (95% CI: −6.5 to −0.5) for FSDS-R, both remaining statistically significant.

Of note, both groups demonstrated a temporal pattern in which most subjective measures peaked at 6 months postoperatively, with a slight decline at 12 months while remaining significantly above preoperative levels.

### 3.6. Objective outcomes

Objective outcome measures, including pelvic floor electromyography (fast‑twitch MVC), LHA, GH, PB, and vaginal capacity, are presented in Table 5. Repeated‑measures analyses revealed significant group‑by‑time interaction effects for pelvic floor EMG amplitude (*P* = .015), LHA (*P* = .018), GH (*P* = .018), and vaginal capacity (*P* = .011), indicating that the full‑length group experienced greater improvements in these parameters over time compared with the non‑full‑length group. No significant interaction was observed for PB (*P* = .120).

**Table 5:** Comparison of objective measurement indicators between the 2 groups before and after surgery.

Indicator	Group	Preoperative	3 mo postop	6 mo postop	12 mo postop	Time effect *P*	Group effect *P*	Interaction *P*
Pelvic floor EMG amplitude (μV) (fast-twitch MVC)	Full-length	28.1 ± 5.9	35.2 ± 5.8*,†	37.8 ± 5.5*,†	36.9 ± 5.7*,†	<.001	.008	.015
	Non-full-length	27.5 ± 6.2	31.5 ± 5.5*	32.0 ± 5.3*	31.8 ± 5.6*			
LHA (cm^2^)	Full-length	22.4 ± 3.8	18.2 ± 3.5*,†	17.5 ± 3.2*,†	17.8 ± 3.4*,†	<.001	.008	.018
	Non-full-length	22.8 ± 3.5	21.0 ± 3.0*	20.8 ± 3.1*	21.1 ± 3.2*			
GH (cm)	Full-length	4.4 ± 0.8	3.1 ± 0.5*,†	2.9 ± 0.4*,†	3.0 ± 0.5*,†	<.001	.021	.018
	Non-full-length	4.5 ± 0.7	3.9 ± 0.6*	3.8 ± 0.5*	3.9 ± 0.6*			
PB (cm)	Full-length	2.8 ± 0.5	3.5 ± 0.5*,†	3.6 ± 0.4*,†	3.5 ± 0.5*,†	<.001	.595	.120
	Non-full-length	2.7 ± 0.6	3.2 ± 0.5*	3.3 ± 0.4*	3.2 ± 0.5*			
Vaginal capacity (fingerbreadths)	Full-length	4.0 (3–5)	2.0 (2–3)*,†	2.0 (2–3)*,†	2.0 (2–3)*,†	<.001*	.002*	.011*
	Non-full-length	4.0 (3–5)	2.0 (2–3)*	2.0 (2–3)*	2.0 (2–3)*			

Pelvic floor EMG amplitude, LHA, GH, and PB were normally distributed; statistical methods are the same as for normal indicators in Table 1. Vaginal laxity score was derived from digital examination grading (normal = 1, mild = 2, moderate = 3, severe = 4) and was non-normally distributed, presented as median (range); repeated measures comparison used GEE.

EMG = electromyography, GH = genital hiatus, LHA = levator hiatus area, MVC = maximal voluntary contraction, PB = perineal body.

* *P* < .05 versus preoperative within the same group.

† *P* < .05 versus full-length group at the same time point.

* *P* values for time, group, and interaction effects for non-normal indicators are based on GEE analysis.

For pelvic floor EMG amplitude, the full‑length group demonstrated significantly greater improvement than the non‑full‑length group at both 6 months (MD = 5.8 μV; 95% CI: 3.66–7.94) and 12 months (MD = 5.1 μV; 95% CI: 2.86–7.34), with the difference favoring the full‑length group. Similarly, the full‑length group showed significantly greater reductions in LHA at 6 months (MD = 3.3 cm^2^; 95% CI: 2.05–4.55) and 12 months (MD = 3.3 cm^2^; 95% CI: 1.99–4.61) compared with the non‑full‑length group. For GH, the full‑length group also achieved significantly greater reduction at 6 months (MD = 0.9 cm; 95% CI: 0.72–1.08) and 12 months (MD = 0.9 cm; 95% CI: 0.68–1.12), both favoring the full‑length group. No significant between‑group difference was observed for PB improvement at any time point (*P* for group effect = 0.595), consistent with the identical perineal body reconstruction procedure performed in both groups.

With respect to vaginal capacity, although both groups achieved a median of 2 fingerbreadths (normal range) postoperatively with no significant between‑group difference at any time point, the full‑length group exhibited a significantly greater overall improvement trajectory (*P* for interaction = .011). Notably, despite both groups reaching the same median vaginal capacity, the full‑length group reported significantly higher VLQ scores (as shown in Table 4), suggesting that subjective perception of tightness is not fully captured by digital examination alone.

Of note, most objective measures in both groups peaked at 6 months postoperatively, with a slight decline at 12 months while remaining significantly above preoperative levels, a pattern consistent with that observed for subjective outcomes.

### 3.7. Vaginal capacity grading

The distribution of vaginal capacity grades at each time point is presented in Table 6. At baseline, no patient in either group had normal vaginal capacity (≤2 fingerbreadths), and the distribution of mild, moderate, and severe laxity was comparable between the 2 groups (*P* = .884).

**Table 6:** Distribution of vaginal laxity grading in the 2 groups (n [%]).

Time point	Group	Normal (2 fingers)	Mild (3 fingers)	Moderate (4 fingers)	Severe (>4 fingers)	*P* value
Preoperative	Full-length	0 (0)	12 (24.0)	28 (56.0)	10 (20.0)	.884
	Non-full-length	0 (0)	10 (20.0)	30 (60.0)	10 (20.0)	
3 mo postop	Full-length	43 (86.0)*	7 (14.0)	0 (0)	0 (0)	.595
	Non-full-length	38 (76.0)*	12 (24.0)	0 (0)	0 (0)	
6 mo postop	Full-length	44 (88.0)*	6 (12.0)	0 (0)	0 (0)	.617
	Non-full-length	39 (78.0)*	11 (22.0)	0 (0)	0 (0)	
12 mo postop	Full-length	43 (86.0)*	7 (14.0)	0 (0)	0 (0)	.595
	Non-full-length	38 (76.0)*	12 (24.0)	0 (0)	0 (0)	

* *P* < .05 versus preoperative within the same group (Wilcoxon signed-rank test).

† *P* < .05 versus control group at the same time point (chi-square test).

At 3 months postoperatively, the proportion of patients achieving normal vaginal capacity (≤2 fingerbreadths) was 86.0% (43/50) in the full-length group and 76.0% (38/50) in the non-full-length group. This proportion was maintained through 6 months (88.0% vs 78.0%) and 12 months (86.0% vs 76.0%) in the respective groups. Within-group comparisons showed that both groups achieved significant improvements in vaginal capacity grades at all postoperative time points compared with baseline (all *P* < .001). However, no significant between-group differences were observed in the distribution of vaginal capacity grades at 3, 6, or 12 months postoperatively (all *P* > .05).

Of note, despite the absence of significant between-group differences in vaginal capacity grading, the full-length group demonstrated a numerically higher proportion of patients achieving normal capacity at each postoperative time point, with a difference of approximately 10 percentage points favoring the full-length group throughout the follow-up period. This trend, combined with the significantly greater improvements in subjective VLQ scores observed in the full-length group (Table 4), further suggests that digital examination alone may not fully capture the superior functional outcomes achieved by the full-length procedure.

## 4. Discussion

Currently, the mucosa-preserving perineal body reconstruction technique centered on narrowing the lower vaginal segment remains a commonly employed procedure for moderate-to-severe VLS.^[5]^ However, this approach has inherent limitations in reconstructing the full axial support structures of the vagina. Although modified full-length tightening concepts have been proposed,^[10–12]^ high-level evidence directly comparing full-length versus non-full-length procedures remains scarce. The present retrospective cohort study was therefore designed to systematically compare the multidimensional efficacy of these 2 surgical approaches.

Our findings demonstrated that the full-length procedure achieved significantly greater improvements in the VLQ, SSQ, total FSFI, and FSDS-R scores compared with the non-full-length procedure (all *P* for interaction <.05). The anatomical basis for this superiority lies in the paradigm shift from “circumferential narrowing of the lower segment to “full-length cylindrical reconstruction,”^[13]^ which restores the normal vaginal axis and luminal morphology,^[14]^ extending the sensation of tightness from the introitus to the entire vaginal length and creating a more uniform encirclement. Notably, although both groups achieved a postoperative vaginal capacity of 2 fingerbreadths (normal range) with no significant between-group difference (*P* = .595), the full-length group still exhibited significantly higher VLQ scores than the control group (*P* = .008). This observation suggests that routine digital examination, once vaginal caliber has normalized, has limited discriminant validity and fails to capture the subjective perceptual differences arising from deeper structural support alterations. We postulate that the full-length procedure, through suspension and reconstruction of DeLancey level I (the uterosacral–cardinal ligament complex),^[4]^ restores vaginal vault depth and axial compliance – a “sense of support” that cannot be measured by transverse caliber alone.^[15]^ This finding further underscores the necessity of adopting a multidimensional evaluation system that integrates both subjective and objective measures in postoperative assessment.

With respect to objective anatomical parameters, the full-length procedure yielded significantly greater reductions in LHA and GH compared with the non-full-length procedure, confirming the necessity of systematic reconstruction across levels I to III.^[9]^ In contrast, no significant between-group difference was observed in PB improvement (*P* = .120), which paradoxically validates the standardization of our surgical protocol – given that the perineal body reconstruction steps were identical in both groups, comparable PB augmentation was expected. The additional reduction in GH and LHA achieved by the full-length procedure can be primarily attributed to the reinforcement of the upper pelvic diaphragm (rectal fascia and levator ani), which exerted a synergistic amplifying effect on the lower compartment. Furthermore, the full-length group demonstrated significantly greater improvement in fast-twitch muscle contraction amplitude compared with the control group (*P* for interaction = .015), suggesting that comprehensive anatomical reconstruction optimizes pelvic floor neuromuscular function and proprioceptive feedback^[16,17]^ – a finding that corroborates the superiority observed in subjective sexual function scores.

From a temporal perspective, most outcome measures in both groups peaked at 6 months postoperatively, with a slight decline at 12 months while remaining significantly above preoperative levels. This trajectory is consistent with the physiological processes of postoperative collagen remodeling and scar maturation (creep effect),^[18]^ suggesting that 6 months represents a critical time point for assessing “optimal efficacy,” whereas the 12-month results more closely reflect the true long-term maintenance level.

In terms of safety, although operative time and intraoperative blood loss were statistically higher in the full-length group, the absolute increments were modest (approximately 5 minutes and 3 mL, respectively) and did not translate into increased complication rates or prolonged hospital stay (*P* > .05). The incidence of dyspareunia was comparable between the 2 groups, confirming the favorable perioperative safety profile of the full-length procedure.

Several limitations of this study should be acknowledged. First, as a single-center retrospective study with a relatively limited sample size, it may have reduced statistical power and introduced potential selection bias. Second, the 12-month follow-up, while sufficient to assess medium-term efficacy, is inadequate to determine long-term stability or the preventive effect against pelvic floor dysfunction over time. Future prospective, large-sample, multicenter randomized controlled trials are warranted to validate our findings and explore the long-term benefits of these surgical approaches. Nevertheless, the well-balanced baseline characteristics (all *P* > .05) and the 100% follow-up rate may have mitigated some degree of selection and information bias inherent to the retrospective design. However, the direction of residual bias – particularly operator bias introduced by the single-center setting – cannot be entirely excluded, and its impact on the effect estimates requires confirmation in multicenter settings.

Finally, the generalizability of our findings should be considered. This single-center study was conducted in a tertiary referral hospital in central China by a highly experienced pelvic floor surgical team, and all patients had moderate-to-severe VLS, which may limit applicability to mild cases, nonspecialized centers, or other populations. Nevertheless, the objective anatomical parameters and validated patient-reported outcome measures provide a reproducible framework for future comparisons across settings. Multicenter studies with diverse patient populations and varying surgical expertise are needed to confirm the external validity of our conclusions.

## 7. Conclusion

In conclusion, this retrospective comparative study demonstrates that, for patients with moderate-to-severe vaginal laxity, extending the surgical repair to the full vaginal length achieves more comprehensive anatomical reconstruction and functional restoration of the pelvic floor support structures, significantly improves sexual function and quality of life, and does not incur additional surgical risks. Therefore, full-length vaginal tightening can be considered one of the preferred surgical options for moderate-to-severe VLS.

## Author contributions

**Conceptualization:** Fengmei Li, Chunyan Song.

**Data curation:** Fengmei Li, Chunyan Song.

**Formal analysis:** Chunyan Song.

**Investigation:** Chunyan Song.

**Methodology:** Chunyan Song.

**Project administration:** Chunyan Song.

**Writing – original draft:** Chunyan Song.

**Writing – review & editing:** Chunyan Song.
